# Catalytic effect of graphene on the inversion of corannulene using a continuum approach with the Lennard-Jones potential

**DOI:** 10.1039/d3na00349c

**Published:** 2023-08-03

**Authors:** Panyada Sripaturad, Amir Karton, Kyle Stevens, Ngamta Thamwattana, Duangkamon Baowan, Barry J. Cox

**Affiliations:** a Department of Mathematics, Faculty of Science, Mahidol University Rama VI Rd Bangkok 10400 Thailand; b School of Science and Technology, University of New England Armidale NSW 2351 Australia amir.karton@une.edu.au; c School of Information and Physical Sciences, University of Newcastle Callaghan NSW 2308 Australia natalie.thamwattana@newcastle.edu.au; d School of Mathematical Sciences, University of Adelaide Adelaide SA 5005 Australia

## Abstract

The catalytic effect of graphene on the corannulene bowl-to-bowl inversion is confirmed in this paper using a pair-wise dispersion interaction model. In particular, a continuum approach together with the Lennard-Jones potential are adopted to determine the interaction energy between corannulene and graphene. These results are consistent with previous quantum chemical studies, which showed that a graphene sheet reduces the barrier height for the bowl-to-bowl inversion in corannulene. However, the results presented here demonstrate, for the first time, that the catalytic activity of graphene can be reproduced using pair-wise dispersion interactions alone. This demonstrates the major role that pair-wise dispersion interactions play in the catalytic activity of graphene.

## Abstract 2

The catalytic effect of graphene on the corannulene bowl-to-bowl inversion is confirmed in this paper using a pair-wise dispersion interaction model.

## Graphical abstract

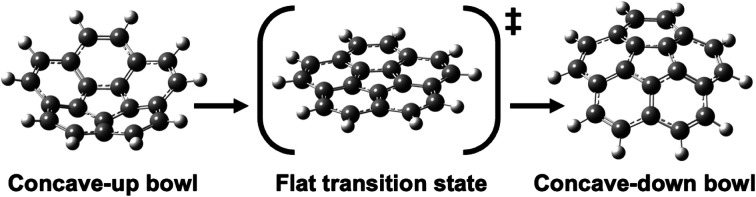

## 1 Introduction

**Fig. 1:**
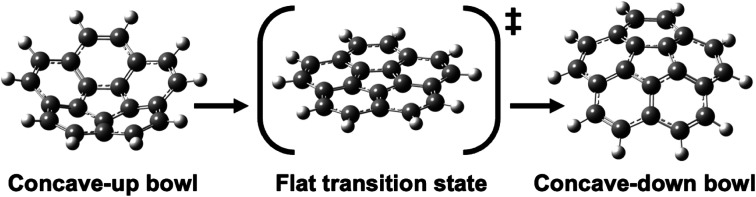
Optimized DFT structures of the equilibrium and transition structures involved in the bowl-to-bowl inversion in corannulene.

Geodesic polyarenes are polycyclic aromatic hydrocarbons in which structural constraints result in a curved π-system.^1–4^ Geodesic hydrocarbons exhibit unique chemical properties, such as large dipole moments and dynamic bowl-inversion behavior.^2–9^ Corannulene (C_20_H_10_) is a prototypical geodesic molecule in which a pentagon surrounded by five hexagons results in a bowl-shaped structure.^10^ Corannulene undergoes a rapid bowl-to-bowl inversion *via* a planar transition structure as illustrated in Fig. 1.^11^ Catalysis of this bowl-to-bowl inversion has attracted considerable attention after it was demonstrated that a cyclophane receptor catalyzes this process *via* induced-fit catalysis.^12–14^ It was later found that graphene (a planar two-dimensional (2D) material composed of sp^2^-hybridized carbons) can also catalyze the bowl-to-bowl inversion in corannulene^15–17^ as well as rotation and inversion reactions in related molecules.^18–20^ It has also been demonstrated *via* extensive density functional theory (DFT) and *ab initio* calculations that these catalytic processes are driven by strong noncovalent interactions that typically exceed a hundred kJ mol^−1^.^15–24^

**Fig. 2:**
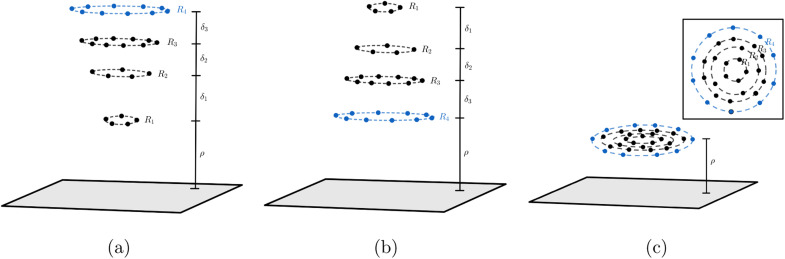
Three conformations of corannulene interacting with graphene sheet: (a) concave-up bowl (b) concave-down bowl (c) flat circular shape. Note that *ρ* is the distance of the closet ring to graphene sheet, and the distances between each ring in corannulene are given by *δ*_1_ = 0.5442 Å, *δ*_2_ = 0.3624 Å, *δ*_3_ = 0.3822 Å.

In the system where graphene is used as a catalyst for corannulene bowl-to-bowl inversion, we envisage that the favourable conformation of corannulene (either concave-up or concave-down bowl) can be determined from the structure that gives rise to the minimum interaction energy with the graphene. Since van der Waals forces dominate the interaction between corannulene and graphene, this paper adopts the Lennard-Jones potential to determine the interaction energy between the two molecules. Here, we assume that carbon atoms on graphene are evenly distributed on its surface so we can use continuum surface approximation to model graphene. For corannulene, its three possible conformations are considered, which are concave-up bowl, concave-down bowl and flat circular structure. Two approaches to model corannulene–graphene interaction are used. The first approach considers corannulene as a collection of 30 discrete atoms (20 carbon and 10 hydrogen atoms), and so the total energy is obtained by summing 30 pairwise interaction energies between each atom on corannulene and a graphene sheet. In the second approach, due to its geometry, we model corannulene as a collection of four circular rings (three carbon rings and one hydrogen ring) centred on the same axis (Fig. 2), and on each ring, atoms are assumed uniformly distributed. As a result, the total interaction energy can be obtained from summing four pairwise interaction energies between each ring and a graphene sheet. For each of corannulene conformations, we find that both approaches give the same energy profile, which is also in agreement with molecular dynamics studies. These results confirm the catalytic effect of graphene on the ability to control the orientation of corannulene that minimises the interaction energy of the system.

In the following section, we give mathematical background for the two approaches to model corannulene–graphene interactions. Detailed calculation of the integrals involved are provided in Appendices A and B. Numerical results for the interaction energies are shown in Section 3 for the three conformations of corannulene. These results are also confirmed by molecular dynamics (MD) and density functional theory (DFT) simulations which their detailed set-ups are given in Appendices C and D, respectively. Finally, concluding remarks is provided at the end of Section 3.

## 2 Interaction energy between corannulene and graphene

Due to its simple form, the Lennard-Jones potential is commonly employed to determine the interaction energy between two non-bonded atoms, which is given by1
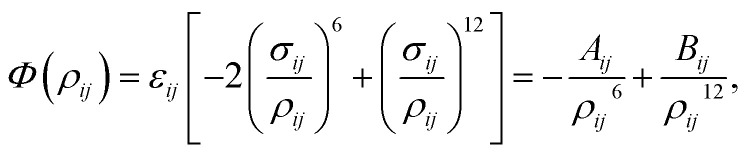
where *ρ*_*ij*_ is the distance between atoms *i* and *j*, *A*_*ij*_ and *B*_*ij*_ are the attractive and repulsive constants, respectively. We note that *A*_*ij*_ = 2*ε*_*ij*_*σ*_*ij*_^6^ and *B*_*ij*_ = *ε*_*ij*_*σ*_*ij*_^12^ where 
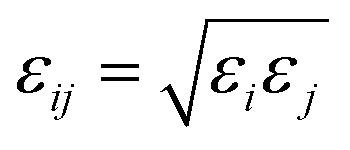
 is the energy well depth and *σ*_*ij*_ = (*σ*_*i*_ + *σ*_*j*_)/2 is the van der Waals diameter. In this paper, the van der Waals parameters for carbon (C) and hydrogen (H) are taken from Rappe *et al.*^25^ where *ε*_C_ = 0.4393 kJ mol^−1^, *σ*_C_ = 3.8510 Å, *ε*_H_ = 0.1841 kJ mol^−1^ and *σ*_H_ = 2.8860 Å. Thus, the Lennard-Jones constants *A*_*ij*_ and *B*_*ij*_ can be evaluated as given in Table 1.

**Table 1:** The attractive and repulsive constants (*A*_*ij*_ and *B*_*ij*_) for carbon–carbon and carbon–hydrogen interactions

Interaction	*A*_*ij*_ (kJ mol^−1^ Å^6^)	*B*_*ij*_ (kJ mol^−1^ Å^12^)
C–C	2865.84	4 673 725.47
C–H	830.93	606 947.72

In a fully discrete approach, the total interaction energy between two non-bonded molecules can be obtained by summing the pairwise potential energy (1) between atom *i* on the first molecule and atom *j* on the second molecule, which is given by2
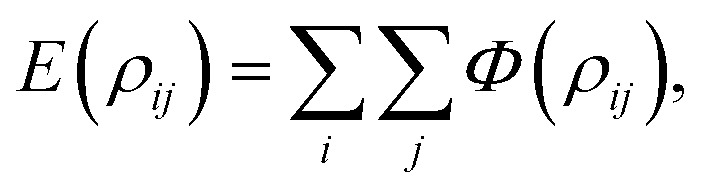
where *ρ*_*ij*_ is the distance between atoms *i* and *j*.

Another method to model the interaction between two non-bonded molecules is known as a continuum approach. This approach assumes that atoms on each interacting molecule are uniformly distributed over its entire surface of the molecule. Thus, the double summation in (2) can be replaced by two surface integrals, namely3
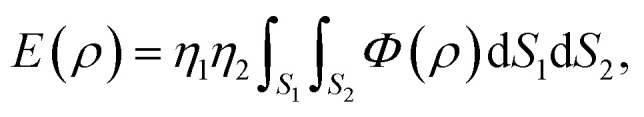
where now *ρ* denotes the distance between typical surface elements d*S*_1_ and d*S*_2_ on the first and second molecules, respectively. The constants *η*_1_ and *η*_2_ are the mean surface atomic densities of the two molecules.

The advantage of using (3) over (2) is the reduction in computational time, especially for large molecules. However, for the integrals in (3) to be traceable to yield analytical expressions, regular shape structures are generally assumed for the interacting molecules. Accordingly, this approach has been commonly adopted to determine the interaction energy involving carbon nanostructures, such as nanotubes, fullerenes, graphene, graphite and nanocones.^26^

In the interest of modelling an irregularly shaped molecule interacting with a regular shaped structure, an alternative hybrid discrete-continuum approach is introduced, which is given by4
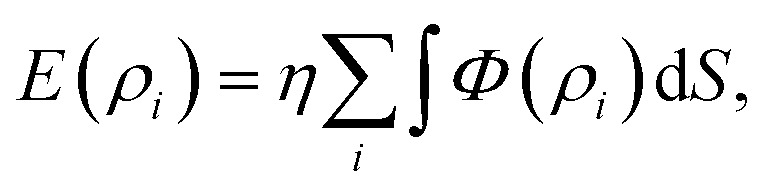
where *η* is the surface density of atoms on the regular shaped molecule, *ρ*_*i*_ is the distance between a typical surface element d*S* on the continuous molecule and atom *i* on the molecule which is modelled as discrete.

In this paper, we use (4) to determine the non-bonded interaction energy between a graphene sheet and a corannulene. Three conformations of corannulene are considered which are depicted in Fig. 2. We model graphene sheet as a continuum flat surface lying on the *xy*-plane. For corannulene, we first assume a fully discrete structure with 30 atoms (10 hydrogen atoms (blue) and 20 carbon atoms (black)) as shown in Fig. 2 (Section 2.1). In Section 2.2, we consider corannulene as a structure comprising four continuous rings, where each ring is arranged as shown in Fig. 2.

### 2.1 Discrete model of corannulene

**Fig. 3:**
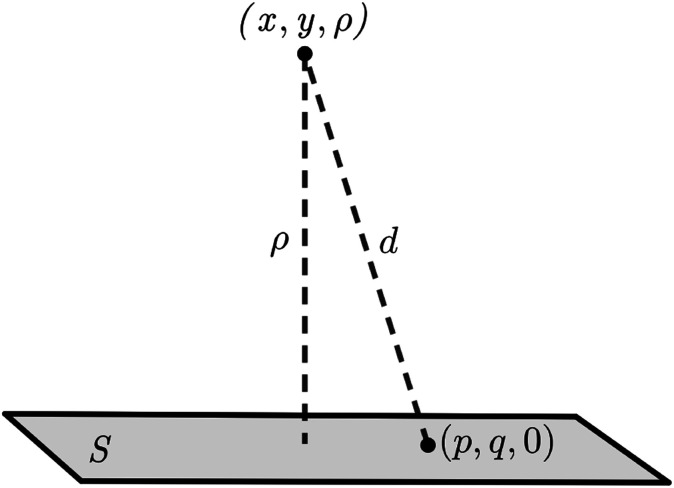
An atom of corannulene located at (*x*, *y*, *ρ*) interacting with an infinite plane of graphene sheet situated on the plane *z* = 0.

Here, we model a corannulene as a collection of 30 discrete atoms. The coordinates of a corannulene in all three conformations can be found from Karton.^15^ Mathematically, we represent an atom on a corannulene as a typical point with coordinates (*x*, *y*, *ρ*) as shown Fig. 3. Each atom then interacts with a flat graphene surface on which a typical point has coordinates (*p*, *q*, 0). Since there are 20 carbon and 10 hydrogen atoms on the corannulene, using (4) the total energy becomes5
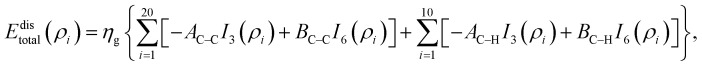
where *ρ*_*i*_ is the vertical distance of atom *i* from graphene sheet, *η*_g_ is the atomic density of graphene sheet (*η*_g_ = 0.3812 Å^−2^) and *I*_*n*_(*ρ*) (*n* = 3, 6) is defined by6
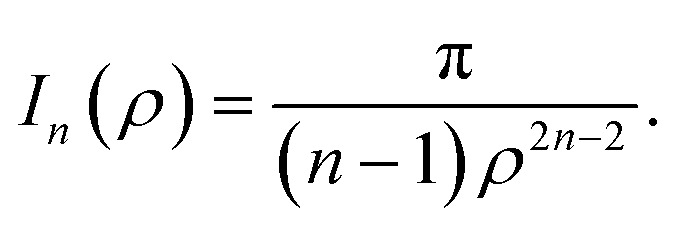


We note that the derivation of *I*_*n*_(*ρ*) is given in Appendix A.

### 2.2 Ring model of corannulene

Since the positions of atoms on a corannulene are as shown in Fig. 2, we assume that these atoms are located on rings R_1_ to R_4_ (see Fig. 2). We note that rings R_1_ and R_2_ each consists of five carbon atoms, ring R_3_ consists of ten carbon atoms and ring R_4_ consists of ten hydrogen atoms. We also note that of these rings, R_1_ involves chemically bonded carbons (*i.e.*, the central pentagon ring of corannulene), whereas R_2_, R_3_, and R_4_ involve non-bonded atoms. These rings are chosen since they are co-planar in the equilibrium (bowl-shaped) and transition state (flat) structures of corannulene. Fig. 2 shows the mathematical representations of the equilibrium and transition state structures of corannulene along with the quantum chemically optimized structures. Further, physical parameters of each ring are given in Table 2.

**Table 2:** Physical parameters of corannulene where *r*_*i*_, *c*_*i*_ and *η*_*i*_ are the radius, circumference and mean atomic density of ring R_*i*_, respectively

	*r*_*i*_ (Å)	*c*_*i*_ (Å)	*η*_*i*_ (Å^−1^)
Carbon ring 1 (R_1_)	1.2108	7.6074	0.6573
Carbon ring 2 (R_2_)	2.4958	15.6818	0.3188
Carbon ring 3 (R_3_)	3.2688	20.5384	0.4870
Hydrogen ring (R_4_)	4.2572	26.7488	0.3738

**Fig. 4:**
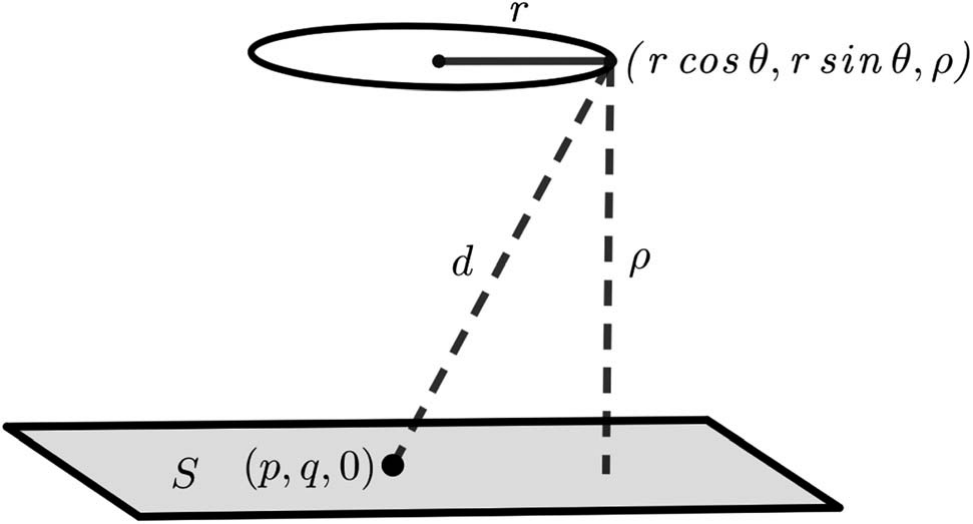
Interaction between a ring of corannulene and a graphene sheet. The graphene sheet is assumed to lie on the plane *z* = 0 and a typical point on a ring of radius *r* situated at a distance *ρ* away from the graphene sheet is given by (*r* cos *θ*, *r* sin *θ*, *ρ*), where *θ* ∈ [0, 2π).

Mathematically, the problem reduces to finding the interaction energy between a ring and a graphene sheet as shown in Fig. 4 and by using (4) we can obtain the total interaction energy as7
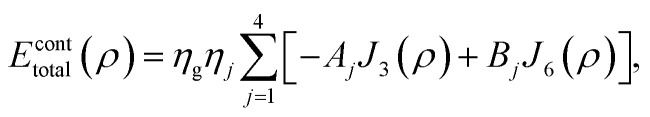
where *η*_*j*_ denotes the atomic density of the ring R_*j*_, *A*_*j*_ = *A*_C–C_ and *B*_*j*_ = *B*_C–C_ when *j* = 1, 2 and 3 and *A*_*j*_ = *A*_C–H_ and *B*_*j*_ = *B*_C–H_ when *j* = 4. The integral *J*_*n*_(*ρ*) (*n* = 3 and 6) is defined by8
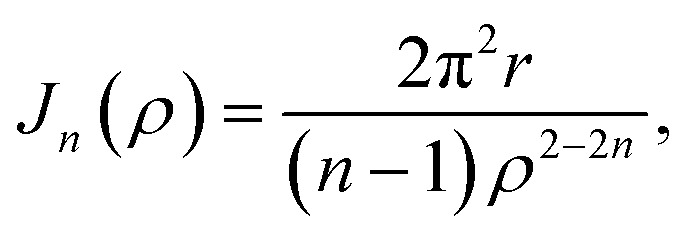
where its derivation is given in Appendix B. As shown in Fig. 2, *ρ* in (7) represents the vertical distance from the graphene sheet to the closet ring of corannulene. For the concave-up bowl, *ρ* is the distance from graphene sheet to ring R_1_ and the distances from graphene sheet to rings R_2_, R_3_ and R_4_ are given by *ρ* + *δ*_1_, *ρ* + *δ*_1_ + *δ*_2_ and *ρ* + *δ*_1_ + *δ*_2_ + *δ*_3_, respectively. Similarly, for a concave-down bowl *ρ* is measured from graphene sheet to the closet ring of corannulene, which is R_4_ in this case. The distances from graphene sheet to rings R_3_, R_2_ and R_1_ are given by *ρ* + *δ*_3_, *ρ* + *δ*_3_ + *δ*_2_ and *ρ* + *δ*_3_ + *δ*_2_ + *δ*_1_, respectively. Finally, for flat circular shaped corannulene, all rings are concentric and have the same vertical distance *ρ* from the graphene sheet.

In the next section, we plot the interaction energies for the three conformations of corannulene interacting with a graphene sheet. The results are also benchmarked with molecular dynamics simulations.

## 3 Results and concluding remarks

**Fig. 5:**
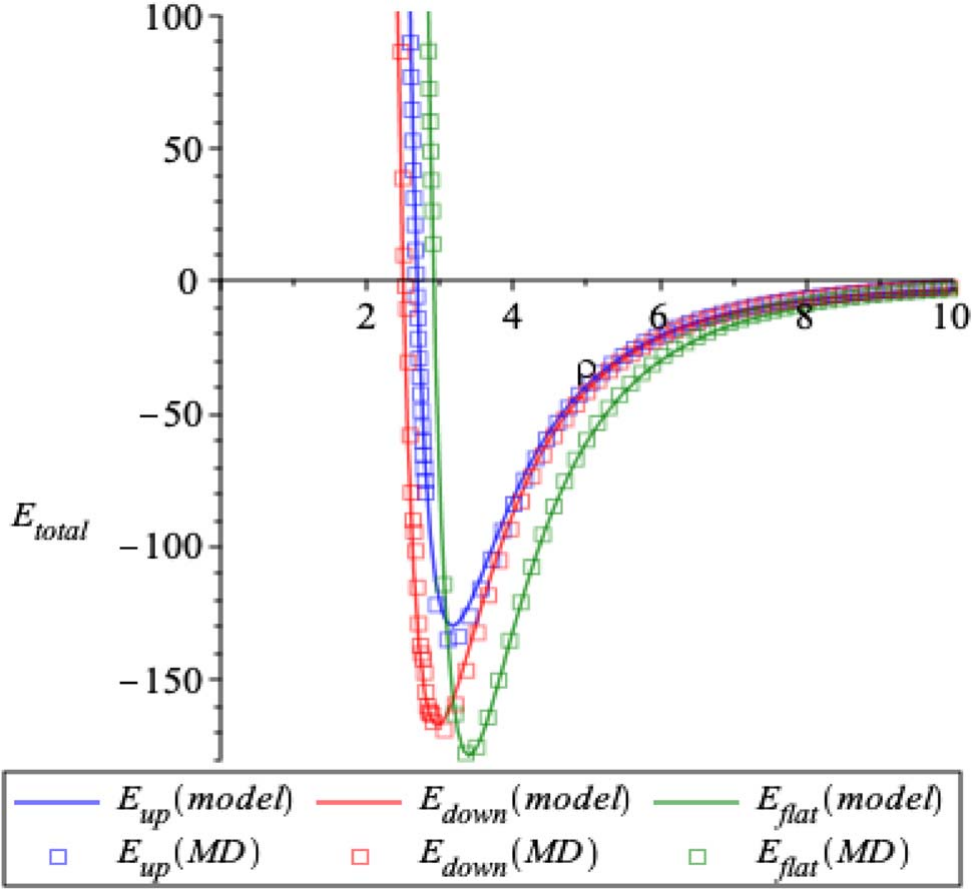
Plot of interaction energy *E*_tot_(*ρ*) (kJ mol^−1^) using our model (solid lines) and molecular dynamics simulations (square boxes) for three conformations of corannulene.

Here, the interaction energy is determined as a function of *ρ* which is the closest distance between corannulene and graphene sheet. We obtain identical results for both discrete and continuous ring approaches, which are plotted as solid lines in Fig. 5 for the three conformations. The results from our model also agree with those of molecular dynamic simulations, which are plotted as square boxes in Fig. 5. From the figure, we can see that there is a preferred distance (*ρ*_min_) for each conformation of corannulene that minimises the interaction energy of the system. The values of *ρ*_min_ and the corresponding minimum energy are given in Table 3. The interaction energy between the planar corannulene transition structure and the graphene sheet amounts to 178.6 kJ mol^−1^, whereas the interaction energy between the concave-up bowl and concave-down bowl and the graphene sheet amount to 129.7 kJ mol^−1^ and 167.3 kJ mol^−1^, respectively. Thus the pair-wise dispersion interactions between the planar graphene sheet and the planar transition structure are stronger by 48.9 and 11.3 kJ mol^−1^, respectively, than the concave-up and concave-down structures. This result is due to the closer proximity of the carbon atoms of corannulene and graphene in the planar transition structure than in the concave-up and concave-down structures.

**Table 3:** The distance *ρ*_min_ and the corresponding minimum energy *E*_total_ obtained from our model and molecular dynamics simulations for corannulene with concave-up bowl, flat circular shape and concave-down bowl interacting with a graphene sheet

Conformation	*E*_total_ (kJ mol^−1^)		*ρ*_min_ (Å)	
	Our model	MD simulations	Our model	MD simulations
Concave-up bowl	−129.663	−135.618	3.163	3.153
Flat circular	−178.597	−178.851	3.390	3.390
Concave-down bowl	−167.288	−170.404	2.959	2.947

The above results are significant since they demonstrate that even in the absence of any explicit quantum chemical interactions, pair-wise dispersion interactions alone would result in a graphene sheet catalyzing the bowl-to-bowl inversion in corannulene. This result is consistent with previous dispersion-corrected, double-hybrid DFT calculations, which were obtained on the Gibbs free potential energy surface.^15,17^ In order to compare on an even keel between the interaction energies obtained using our pair-wise dispersion model we need to calculate the DFT interaction energies on the electronic potential energy surface. For this purpose, we performed DFT calculations on the electronic potential energy surface using the PW6B95-D4 functional (see Appendix D for further details). At the PW6B95-D4/def2-TZVPP level of theory with basis set superposition error (BSSE) corrections, we obtain the following interaction energies between corannulene and graphene 82.6 (concave-up bowl), 111.3 (planar TS), and 87.1 (concave-down bowl) kJ mol^−1^. Using a larger quadruple-*ζ* basis set without BSSE corrections, namely at the PW6B95-D4/def2-QZVPP level of theory, we obtain similar interaction energies of 87.1 (concave-up bowl), 117.1 (planar TS), and 91.6 (concave-down bowl) kJ mol^−1^. There is little to choose between the two levels of theory since both have different advantages and disadvantages in terms of basis-set completeness. However, the differences of 4.4–5.8 kJ mol^−1^ between both sets of results indicate that we are only a few kJ mol^−1^ away from the complete basis set limit. Here, we will focus on the results obtained with the larger def2-QZVPP basis set. The pair-wise dispersion model predicts much larger interaction energies of 129.7 (concave-up bowl), 178.6 (planar TS), and 167.3 (concave-down bowl) kJ mol^−1^ (Table 3). However, due to the systematic overestimation of the interaction energies for the concave-up, planar, and concave-down complexes, the catalytic enhancements predicted by the pair-wise dispersion model are in reasonable agreement with the PW6B95-D4/def2-QZVPP results. In particular, the pair-wise dispersion model predicts catalytic enhancements of 48.9 and 11.3 kJ mol^−1^ for the forward and reverse directions, whereas the PW6B95-D4/def2-QZVPP level of theory results in catalytic enhancements of 30.0 and 25.5 kJ mol^−1^ for the forward and reverse directions. We note that the smaller interaction energies obtained in the DFT simulations are partly attributed to the use of a C_96_H_24_ graphene nanoflake model. We expect that using larger graphene nanoflake models would result in larger interaction energies (for further details, see ref. 23). We also note that the pair-wise dispersion model and DFT interaction energies both suggest that the concave-down complex is energetically more stable on the electronic potential energy surface, albeit the DFT results suggest a smaller energy difference between the concave-up and concave-down complexes. Overall, these results demonstrate that pair-wise dispersion interactions play a major role in the catalytic activity of graphene.

## Appendix A evaluation of integral *I*_*n*_ in section 2.1

Here, we consider a typical point (*x*, *y*, *ρ*) as a location of an atom on corannulene interacting with a graphene sheet lying on the *xy*-plane on which its typical point has coordinates (*p*, *q*, 0). To determine the interaction energy between a single atom and a graphene sheet using the Lennard-Jones potential, we introduce the integral *I*_*n*_(*ρ*) which is given as9
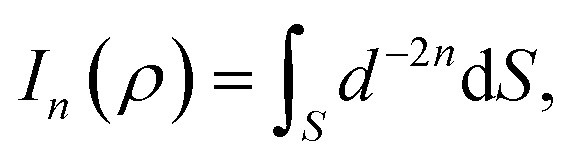
where d*S* is the surface element of graphene sheet and *d* is a typical distance between atom on the corannulene and graphene sheet (Fig. 3) such that*d*^2^ = (*x* − *p*)^2^ + (*y* − *q*)^2^ + *ρ*^2^.

Since we assume that the size of graphene is much larger than the dimension of a corannulene, we can model graphene as an infinite plane. Thus, (9) can be written as10
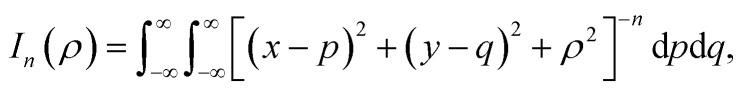
where *n* = 3, 6. Let *K* = *p* − *x* and *M* = *q* − *y* so (10) is reduced to11
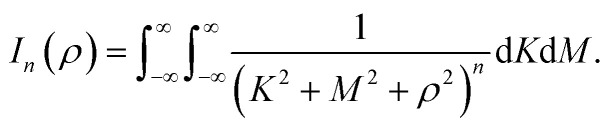


Next, we substitute 
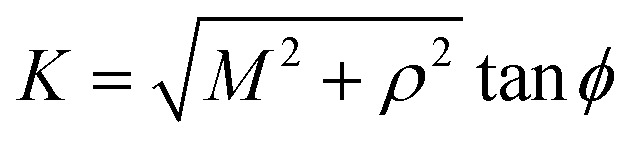
, where *ϕ* ∈ (−π/2, π/2) so that (11) becomes12
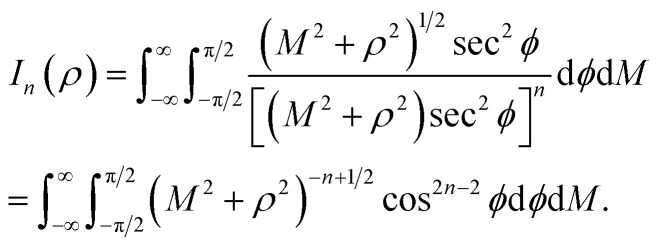


Since
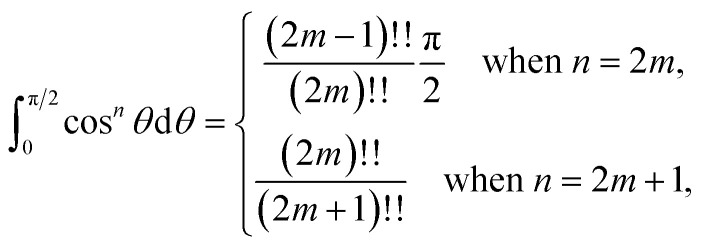
consequently, (12) can be written as
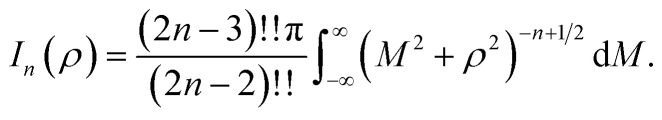


By further substituting *M* = *ρ* tan *ψ* where *ψ* ∈ (−π/2, π/2), we obtain
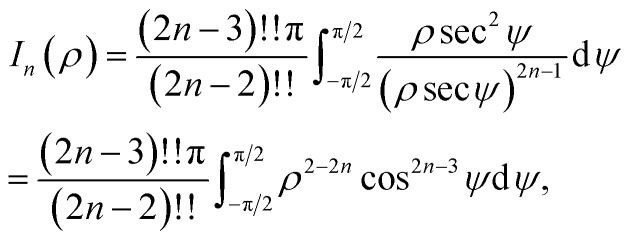
which yield
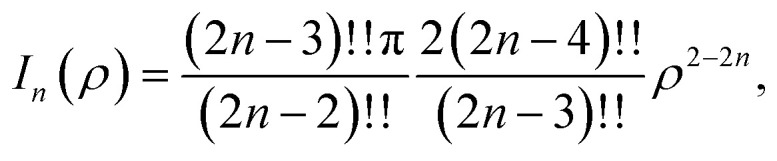
and upon simplifying gives (6).

## Appendix B evaluation of integral *J*_*n*_ in section 2.2

Here, we consider a ring of radius *r* with typical point (*r* cos *θ*, *r* sin *θ*, *ρ*) interacting with a graphene sheet lying on the *xy*-plane on which its typical point has coordinates (*p*, *q*, 0). To determine the interaction energy between a ring and a graphene sheet using the Lennard-Jones potential, we introduce the integral *J*_*n*_(*ρ*) which is defined by13
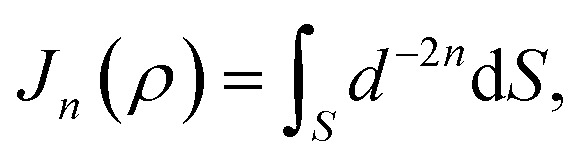
where d*S* is the surface element of graphene and *d* is the distance between typical points on the ring and graphene sheet (Fig. 4), which is given by*d*^2^ = (*r* cos *θ* − *p*)^2^ + (*r* sin *θ* − *q*)^2^ + *ρ*^2^.

Thus we may write (13) as14
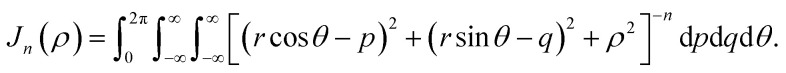


Now let *W* = *p* − *r* cos *θ* and *T* = *q* − *r* sin *θ*, hence (14) becomes
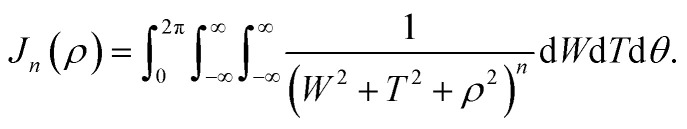


By noting that 
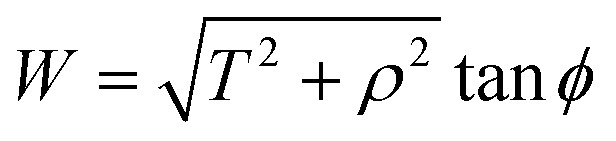
 and *T* = *ρ* tan *ψ* and following the same procedure as in Appendix A, we find
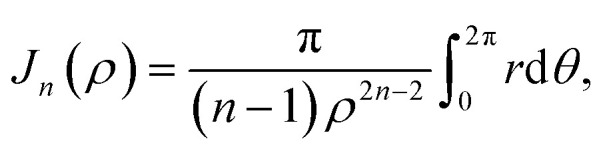
and hence, we obtain (8).

## Appendix C molecular dynamics simulations

The large-scale atomic/molecular massively parallel simulator (LAMMPS) software package^27^ was used to perform the simulations for the corannulene–graphene interaction. Results of these simulations are reported in Section 3. The system is simulated in a domain of size 100 Å × 100 Å × 100 Å. The Lennard-Jones pair potential is used with a cut-off distance of 14 Å and only atomic coordinates are considered, no bonds, angles nor dihedrals. To compare simulation results with the models mentioned in Section 2, the corannulene is forced to move along the *z*-axis as opposed to allowing the program to determine the movement from a set of initial conditions. The corannulene molecule is also rotated about its own axis during the run time in order to minimise effects from configuration bias, since the ring model it is being compared to does not account for this. This method of simulation is equivalent to numerical solution where corannulene is modelled as a collection of discrete atoms.

## Appendix D density functional theory simulations

Density functional theory (DFT) calculations were performed using the hybrid meta-GGA (generalized gradient approximation) DFT method PW6B95-D4 in conjunction with the triple- and quadruple-*ζ* def2-*n*ZVPP basis sets (*n* = T, Q).^28,29^ Where the recently developed, atomic-charge dependent D4 dispersion correction is employed.^30,31^ The PW6B95 exchange-correlation functional has been extensively benchmarked and found to be robust for both reaction energies and barrier heights involving related systems.^18,20,32–38^ We were able to perform basis set superposition error (BSSE) calculations in conjunction with the def2-TZVPP basis set.^39–41^ However, the BSSE calculations in conjunction with the def2-QZVPP basis set proved beyond our computational resources. All optimized geometries were taken from ref. 17. All calculations were carried out using the Gaussian 16 program suite.^42^

## Conflicts of interest

There are no conflicts of interest to declare.

## Supplementary Material
